# Traffic-Weighted Detour Ratio Identifies Inefficient Cycling Routes

**DOI:** 10.3390/e28060670

**Published:** 2026-06-11

**Authors:** Xinze Qiu, Tianli Gao, Jingru Yu, Jianying Wang, Yongping Zhang, Ruiqi Li

**Affiliations:** 1 Hangzhou International Innovation Institute, Beihang University, Hangzhou 311115, China; 2 Institute of Nuclear and New Energy Technology, Tsinghua University, Beijing 100084, China; 3 School of Information Engineering, Xi’an Mingde Institute of Technology, Xi’an 710124, China; 4 Thrust of Urban Governance and Design, Hong Kong University of Science and Technology (Guangzhou), Guangzhou 511453, China; 5 School of Public Affairs, Zhejiang University, Hangzhou 310058, China; 6 ZJU-CMZJ Joint Lab on Data Intelligence and Urban Future, Zhejiang University, Hangzhou 310058, China

**Keywords:** dockless sharing bikes, cycling-traffic-weighted detour ratio, infrastructure, vehicle traffic

## Abstract

Urban congestion is simultaneously influenced by heterogeneous spatio-temporal travel demands, the topology and spatial characteristics of road networks, and the interplay between multiple travel modes. As a critical component of solutions towards a greener and more sustainable transportation, bike-sharing systems have great potential in reducing carbon emissions, improving public health, and alleviating congestion by substituting short-distance motorized trips. Benefiting from flexible accessibility and usage, dockless bike-sharing has gained wide popularity and revived the fashion of cycling in cities. In this study, we reveal that the widely adopted detour ratio alone cannot effectively reflect congestion levels at the route level. Using large-scale dockless bike-sharing data and taxi trajectory data in Beijing, we quantitatively examine the relationships between cycling flow, motor vehicle traffic and road network structure. In addition, the proposed cycling-traffic-weighted detour ratio can prescreen potentially inefficient cycling routes, which can assist targeted infrastructure optimization and evidence-based urban planning.

## 1. Introduction

Most modern cities have followed a car-centric development since the 20th-century [1], and the booming of cars and car-orientated transportation systems has led to a variety of urban illnesses, including traffic congestion, air pollution, energy deficiency, and deterioration of public health [2,3,4]. Though having an ideal of providing a solution to sustainable mobility, at least in the United States, car-sharing platforms have intensified traffic congestion in both intensity and duration since their launch [5]. Over the past few decades, with growing concerns over global warming and rapid urban sprawl [6], numerous efforts have been devoted to promoting public bike-sharing systems in cities as a greener, more resilient and healthier mobility solution to reduce carbon emissions and improve public health [4,7,8,9,10]. During the COVID-19 pandemic, bike-sharing systems proved to be more resilient [11] and safer to move around for essential needs, since cycling allows for greater social distancing than other means of public transportation [12,13,14]. With great advances in IoT (Internet of Things) and mobile payment technology, and attributed to their better affordability [15], accessibility and usage flexibility [16], newly emergent dockless sharing bikes [12,17,18] have become quite popular and revived the cycling fashion, with dozens of millions of cycling trips over hundreds of cities [12].

It is commonly believed that cycling has potential for replacing short-distance motorized trips [19,20] with a myriad of benefits to both individuals and the whole city, including increasing fitness and reducing the stress of riders from cycling activities [20,21,22,23], saving parking space and fossil energy [24,25], and mitigating urban congestion [26]. Previous evidence also indicates that promoting public bike-sharing systems can reduce car usage [3,19], and increase the share of public transportation via covering the last mile more easily by cycling [20,27,28]. However, the interplay between travel modes (e.g., driving and cycling) and congestion is complex and still unclear. Urban congestion and transportation efficiency are simultaneously influenced by the topology and spatial characteristics of road networks [29] together with heterogeneous spatio-temporal travel demands [30]. If inefficient routes, which are of high detour distance, are used by many people, they will have stronger impacts on urban congestion and transportation efficiency [30]. In addition, different travel modes might have influence over each other. However, there have been few works considering all the above factors in a cohesive way for gaining a better understanding of urban congestion.

In this work, by exploiting massive cycling activities via dockless sharing bikes and detailed vehicle congestion data over six diversified cities in China—Shanghai, Beijing, Ningbo, Xiamen, Yiwu, and Lishui—we first discover that the distributions of detour ratios on cycling in different cities are quite similar, and the detour ratio is negatively correlated with Euclidean distance over cities. The detour ratio, which equals the shortest routing distance on road networks over Euclidean distance between locations, reflects how many additional detours are needed to get from one place to another [29,30]. The sum of detour ratios has been widely adopted as a measure of transportation efficiency in transportation studies [31,32] with a common assumption that the greater the detour ratio, the lower the efficiency of the transportation process. However, from the empirical analysis across cities, we indeed discover that the detour ration for each route is uncorrelated with its motor traffic congestion severity. This can be a consequence of the complex interplay between cycling traffic and vehicle traffic; the two would highly probably influence each other, especially when there are no dedicated biking lanes or when road conditions are complex. Recent advances have also pointed out that cycling route choice can be greatly affected and largely driven by user perceptions on the complexity of routes [33,34], the safety level of routes, and also the built environment [35], rather than merely the detour ratio, travel distance, or congestion level. Therefore, the traditional detour ratio alone can be insufficient to capture real-world route inefficiency experienced by cyclists. In addition, the topology and spatial characteristics of road networks would also play a part. With taxi trajectory data from Beijing, we further explore the relation between cycling flow, vehicle traffic, and road structure. Eventually, the cycling-traffic-weighted detour ratio is applied to detect inefficient cycling routes.

The rest of the paper is organized as follows. Section 2 presents some basic analysis of characteristics of cycling behaviors via dockless sharing bikes. Section 3 shows the distribution of detour ratios for cycling trips during rush hours and nonpeak hours over different cities, and explores the relationship between the detour ratio and Euclidean distance. Section 4 reveals that the detour ratio alone cannot reflect traffic congestion for routes, and further analyzes cycling flow, vehicle traffic, and road structure to reveal their underlying relation. Section 5 identifies inefficient routes via the cycling-traffic-weighted detour ratio. Section 6 concludes the work, points out limitations, and highlights future research directions.

## 2. Data and Methodology

In this study, we collect 663 million cycling trips with dockless sharing bikes in six diversified cities in China—Shanghai, Beijing, Ningbo, Xiamen, Yiwu and Lishui—which have varying population sizes and different urban terrains. The cities included in this study were selected based on three key criteria: geographical location, population size, and bike-sharing operation scale. With diversified cities under scrutiny, the representativeness and generality of the results can be better guaranteed. In addition, the datasets in this study span several years, covering periods of fast expansion and stable operation of dockless bike-sharing platforms [18], and even cover periods affected by the COVID-19 pandemic. If datasets can be collected in the same year and from the same platform, we can better control other factors that might affect cycling activities across cities. Yet, as we will show later, most patterns remain stable in different years and across diversified cities.

Basic information and statistics of datasets, including the platform, period, the numbers of orders, users, and bikes, urban population, urban area with cycling activities, and GDP, are given in Table 1. The Shanghai and Beijing datasets are obtained from the Mobike platform (Beijing, China). The Ningbo, Yiwu and Lishui datasets are obtained from the Hellobike platform (Shanghai, China). The Xiamen dataset is aggregated from the three largest platforms in the city—Meituan Bike (Beijing, China) (formerly Mobike), Hellobike and DiDi Bike (Beijing, China). Obtaining data from different platforms can also better guarantee the generality of the findings in this work. The datasets are formatted as follows: an order ID, an anonymized user ID, a bike ID, a start time and end time, a start position and an end position in (latitude, longitude). The Beijing dataset does not have the end time of each trip, and we estimate it by making queries via the Amap API to obtain the shortest route and its corresponding travel time (https://lbs.amap.com, and Amap is the Chinese counterpart of Google Map), so we are unable to obtain the actual cycling velocity for the Beijing dataset. Different from other cities’ datasets, which contain the data for the whole day, the Xiamen dataset is limited to 6 a.m. to 10 a.m. of each day and does not have the user ID for each record due to privacy and business concerns. To make a better traffic analysis, we rasterize the urban space into 500 m × 500 m grids (i.e., locations) for Shanghai and Beijing and 300 m × 300 m grids for Ningbo, Xiamen, Yiwu and Lishui, as Beijing and Shanghai are megacities, while other cities are prefecture-level or even county-level cities (e.g., Yiwu), which are small for both population size and spatial scale. Then we associate each start and end position to rasterized locations.

For the raw data, we do some simple filtering: we discard the records with origin or destination not located within the boundary of the city and also discard the ones with a cycling duration longer than one day (which might be some bikes left unlocked after their initial order) or less than one minute (which might be due to unsatisfied tryouts). To avoid possible biases, we do not pose any further filtering criteria.

**Figure 1:**
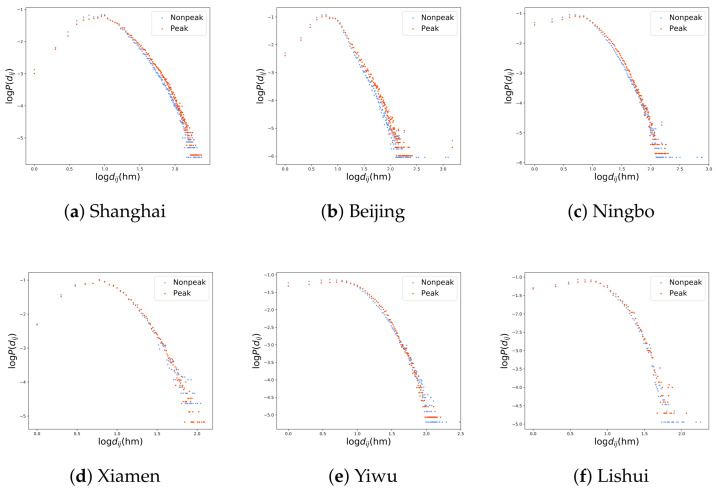
Distribution of Euclidean cycling distance in six cities.

**Figure 2:**
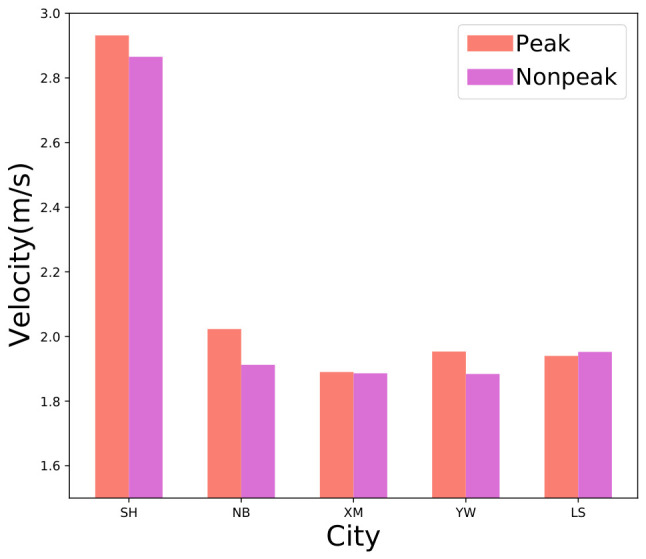
Average cycling velocity in cities. Cities are ordered by urban population, and as the Beijing dataset does not provide the actual arrival time of each trip, it is not shown in this figure.

**Figure A1:**
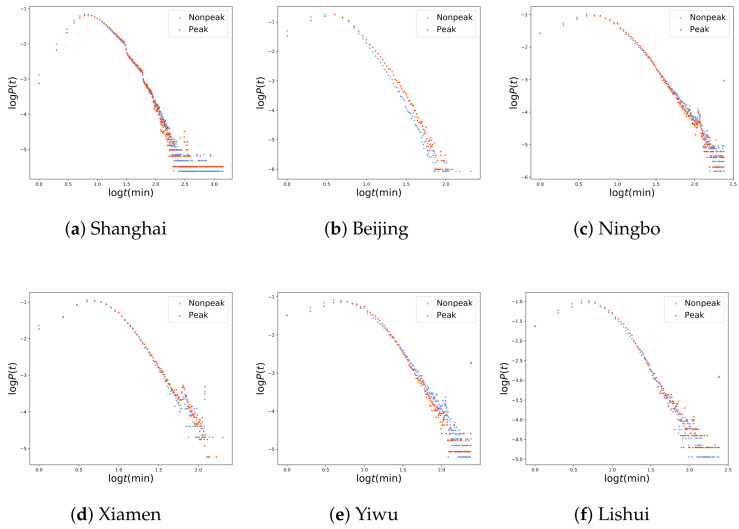
Distribution of cycling duration in cities.

The Euclidean distance of trips by dockless sharing bikes in cities can be well approximated by a log-normal distribution (see Figure 1), which peaks at around 1 km but has a non-negligible fraction of trips with much longer displacements regardless of whether they are during rush hours or nonpeak hours. And it is worth noting that there are more trips with a longer distance during rush hours than during nonpeak hours in Shanghai, Beijing and Ningbo. This pattern tends to happen in big cities, which indicates that for commuting, people may have to ride bicycles for longer distances to save time (see Figure A1) or to avoid crowding in public transportation, and this happens mainly for trips roughly longer than two kilometers and shorter than ten kilometers. Such a pattern is different from the taxi, which tends to have a shorter trip distance and duration during rush hours than during nonpeak hours [36]. In addition, some recent evidence also indicates that cycling is of the highest quality of commuting experience when compared to driving, taking buses, and walking [37]. Meanwhile during nonpeak hours, when trips are not that urgent (e.g., sightseeing or exercise), people have a weaker tendency to cycle for relatively long trips (see Figure 1). Here, peak hours are from 7 a.m. to 9 a.m. and 5 p.m. to 8 p.m., and other time slots are nonpeak hours. As the Xiamen dataset is from 6 a.m. to 10 a.m., the results shown in Figure 1d are limited to this time period, which also explains why the distribution of displacement distance is similar there. From the patterns shown in Figure 1 and Figure A1, we speculate that people use dockless sharing bikes mainly for short trips to get around the neighborhood quickly [20,27,28] or to connect commuting [38], but also ride them for long trips relatively more frequently than a normal or Poisson distribution would assume. In terms of cycling velocity, people generally ride faster during peak hours than during nonpeak hours (see Figure 2). This is because the purpose for people riding bikes during peak hours is saving time for commuting. The average cycling speed in Shanghai is greater than that in other cities, which also echos the finding in urban scaling law that larger cities have a faster life pace [39,40]. Some evidence indicates that users at the fringe of cities usually have a long average travel distance with dockless sharing bikes, which indicates that sharing bikes serve as more than just short-distance commuting connections there [16] or a less developed public transportation there.

**Figure 3:**
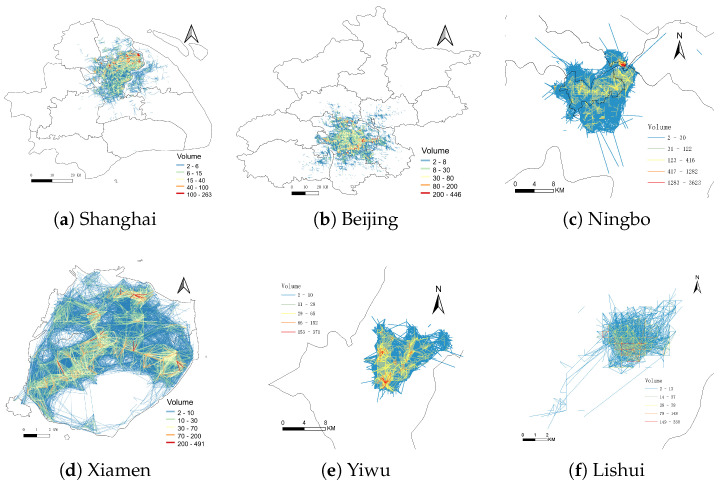

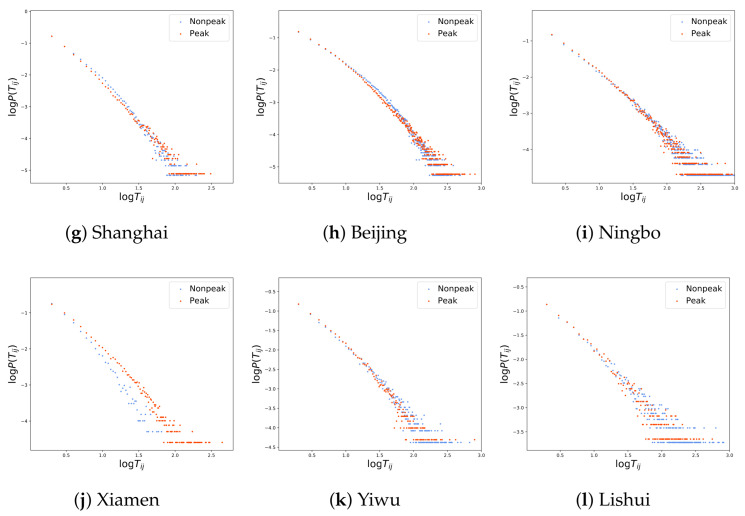
Distribution of cycling traffic in cities. For better clarity, cycling traffic between location pairs that equals one is not shown in (**a**–**f**), the complete cycling traffic distributions are shown in (**g**–**l**).

When aggregating each trip to locations, we obtain the origin–destination matrix of cycling traffic in cities (see Figure 3). Cycling traffic in the peripheral regions of the city is generally low, but location pairs generating larger cycling flow are also relatively dispersed, which are near dense residential communities and workplaces. Apart from being influenced by population [16], bicycle traffic is also constrained by geographical or hydrological factors; for example, there are quite a few trips crossing the Huangpu River in Shanghai (see Figure 3a) or the Xiamen Botanical Garden and Dongping mountain (see Figure 3d). Despite varying geographical characteristics across cities, the distributions of cycling traffic all follow a power-law during both rush hours and nonpeak hours (see Figure 3g–l). This finding indicates a strong heterogeneity of cycling flows that most routes have low bicycle traffic but there is also a notable fraction of routes that have high bicycle traffic, which takes up a large fraction of cycling traffic in the whole city (see Figure 3a–f).

## 3. Road Network Topology and Detour Ratio of Cycling

**Figure 4:**
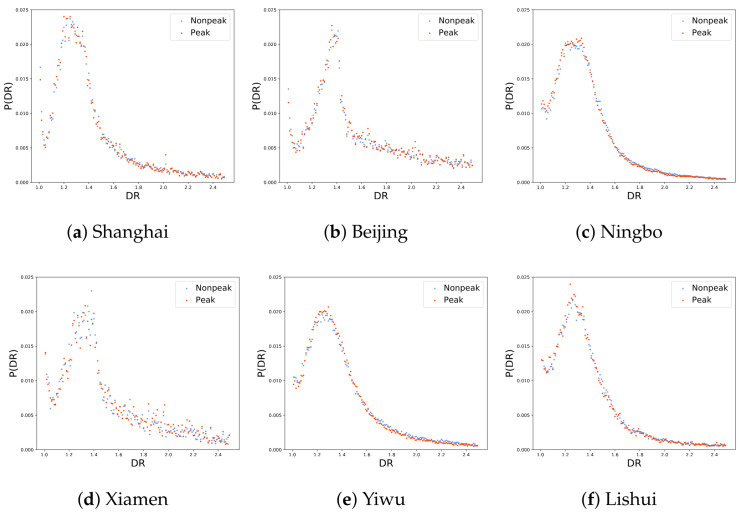
Distribution of detour ratio for cycling trips in six cities.

In traffic engineering and geographical analysis of human activities, the detour ratio (DR) [29,30,32,41,42] has been widely applied for assessing transportation efficiency, and recent advances also indicate a strong relation between DR and navigation cognitive ability of residents [33], which has great impacts on both cycling activities and vehicle traffic. The DR equals the actual routing distance $r_{ij}$ on the road network over the Euclidean distance $d_{ij}$ between two locations *i* and *j*, which is formulated as

(1) $$DR_{ij}=\frac{r_{ij}}{d_{ij}},$$

where $r_{ij}$ is queried from the Amap API for the fastest routing path for each trip by car, $d_{ij}$ is from the great-circle distance between the start and end locations, and the value of $DR_{ij}$ is larger than or equal to one. Here, we only consider location pairs with cycling traffic. The distributions of DR for location pairs with cycling activities during both peak hours and nonpeak hours are almost identical (see Figure 4), which indicates that the spatial patterns of cycling trips are quite similar during different times.

**Figure A2:**
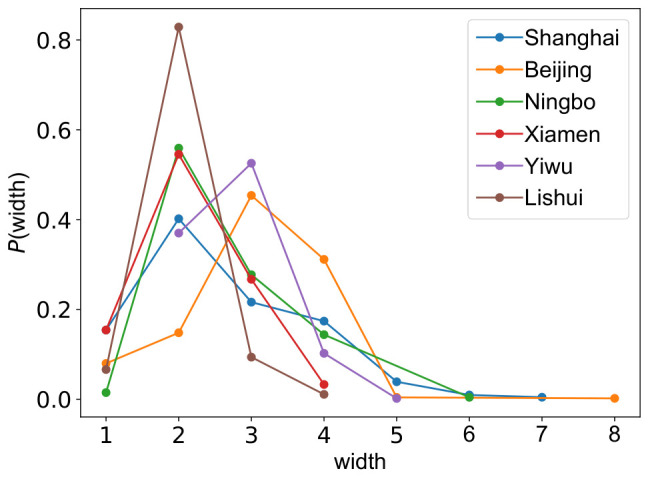
Distribution of road width (the number of lanes) in six cities. The road network data is obtained from OpenStreetMap [47].

In general, DR peaks around 1.3 to 1.4 and exhibits similar distributions across cities, which further reflects potential universal regularities behind the interplay between the topology and geographic structure of road networks [29]. DR quantifies physical routing inefficiency complementary to safety, comfort, and infrastructure supply. A higher value of the sum of DR across routes generally corresponds to lower transportation efficiency [31,32]. In addition, we can observe that the distributions of DR in Beijing and Xiamen are more right-skewed than those in other cities (see Figure 4) and the median DRs of Beijing and Xiamen are also larger than in other cities (see Table 2), which might be due to a larger block size [43] and segmenting effects posed by wider roads (e.g., in Beijing, the 2nd to 5th Ring Roads are highways located within the urban area; when there is no pass-through near highways, it would cause long detours) or a more complex urban terrain (e.g., in Xiamen, there are several mountains and lakes located in the urban area). In Chinese cities, gated communities [44], gated working units and gated universities are quite common, and they generally further make trips more detoured, as outside traffic is usually unable to pass through and has to detour when encountering such enclosed regions. In Beijing, the block size, as well as road width (see Figure A2), is usually larger than other cities. It is worth noting that the improvement priority of detoured routes should also depend on the travel demands on them. If a very detoured route is used by many people for commuting, then it should be improved with a higher priority, while, if only a few people are using it, then it will have a milder impact.

**Figure 5:**
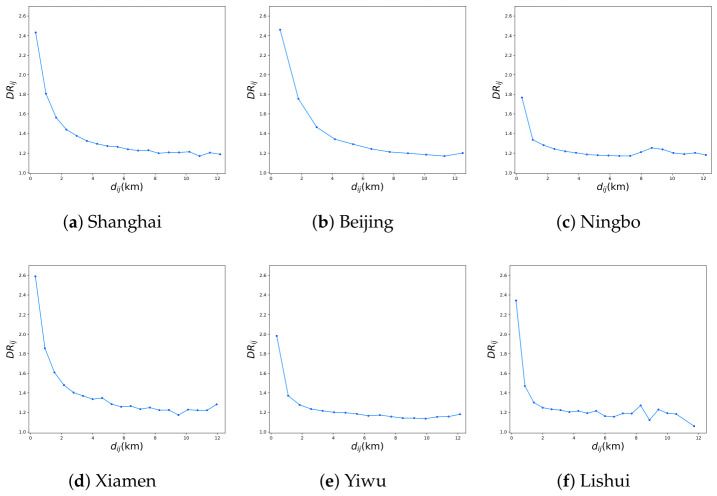
Detour ratio against Euclidean distance for cycling trips in six cities.

We find that the DR shows a nonlinear negative correlation with the Euclidean distance in all six cities (see Figure 5), and most roads with large DR generally have Euclidean distances less than a few kilometers. As the Euclidean distance increases, the detour ratios all tend to be around 1.2. It is noticed that Beijing and Xiamen have higher detour ratios in the roads with smaller Euclidean distances than in other cities, which is consistent with the previous findings.

The existence of one-way roads [29], where people can only drive or cycle in one direction, would also result in a higher DR. In practice, bidirectional cycling traffic may exist even on one-way roads, and this would usually lead to disturbances to pedestrians or normal vehicle traffic due to a higher chance of dangerous encounters between vehicles and bikes.

## 4. Interplay Between Travel Modes and Road Networks

**Figure 6:**
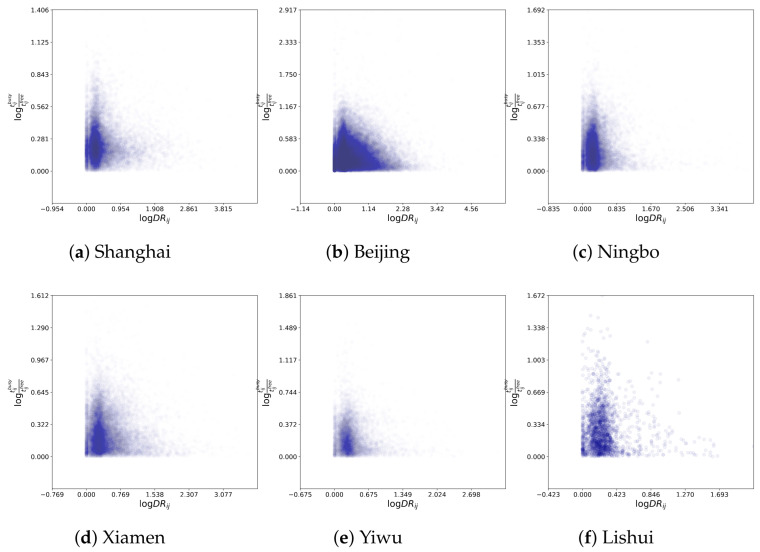
Traffic congestion is uncorrelated with detour ratio at location pair level.

To further analyze the relation between urban congestion and cycling traffic and road network structure, we extract the data during morning rush hours (6:00 a.m. to 10:00 a.m.) on working days in all six cities. From the Amap API, we further query the vehicle travel time (i.e., driving time) for each location pair during rush hours $t_{ij}^{busy}$ and free-flow travel time at midnight $t_{ij}^{free}$ to quantify traffic congestion on the shortest path connecting locations *i* and *j*:

(2) $$congestion_{ij}=\frac{t_{ij}^{busy}}{t_{ij}^{free}},$$

whose value is larger than or equal to one. DR has long been applied for evaluating transportation efficiency; here, we analyze the relation between DR and traffic congestion. However, we discover that at the location pair level, DR alone is almost uncorrelated with traffic congestion (see Figure 6). This might be a consequence of the complex interplay between heterogeneous spatio-temporal travel demand, road capacity and spatial layout road network, and the choice of travel modes. For example, vehicles and cyclists on the same route would highly probably influence each other especially when there are no dedicated biking lanes or when road conditions are complex. In addition, how detoured a route is would also play a part in affecting the behaviors of drivers and cyclists. We assume that the traffic congestion $congestion_{ij}$ should be a function of the detour ratio $DR_{ij}$, capacity of roads $C_{ij}$, vehicle traffic $V_{ij}$ and cycling traffic $T_{ij}$, which reflect the choice of travel modes, i.e., $congestion_{ij}=f\left(DR_{ij},C_{ij},V_{ij},T_{ij}\right)$.

The traffic congestion on a certain route was assumed to be related to the demand over supply [45,46] (i.e., traffic volume over road capacity), which is generally formulated as the Bureau of Public Roads (BPR) function ${\left(1+\eta \left(V_{ij}/C_{ij}\right)\right)}^{\beta }$. Cycling traffic and the detour ratio may also play a part in traffic congestion; we assume that

(3) $$\ln congestion_{ij}=\ln\frac{t_{ij}}{t_{ij}^{free}}=\beta \ln\left(1+\eta \left(\frac{V_{ij}}{C_{ij}}\right)\right)+\alpha \ln T_{ij}+\gamma \ln DR_{ij},$$

where $t_{ij}$ is the travel time by car given the vehicle traffic $V_{ij}$ and road capacity $C_{ij}$; η and β are coefficients that can be better estimated from empirical data. Here, $C_{ij}$ can be estimated from metadata related to roads (e.g., the number of lanes, speed limit, road category) [47].

Here, we use traffic of taxi $V_{ij}$ in Beijing in the year 2017 (the taxi data is 1–7 March 2017; although two months ahead of the biking data in Beijing, it is the closest one that we can obtain), and estimate the road capacity $C_{ij}$ with the road network from OpenStreetMap [47] to perform regression analysis. By regression analysis, we obtain that β=1.20,α=−0.03, and γ=−0.05, with $R^{2}=0.62$. This indicates that a larger volume over capacity leads to heavier congestion, while a larger cycling traffic would ease it, and DR also mitigates the congestion. This might be due to route avoidance behaviors and urban road network hierarchy effects in that drivers of motor vehicles might tend to avoid very detoured or congested routes, and thus partially contribute to a negative relation between motor traffic and cycling traffic. In addition, the impacts of the structure of road networks are also entangled; for example, for very detoured and low-capacity routes, cycling might indeed be a better choice than driving [20]. Furthermore, public transit can also play an important role, which was excluded in this study due to the inaccessibility of such data. Once the full sample vehicle traffic data and public transit data are available in Beijing or in other cities, the validity and universality of such a relation can be better tested.

## 5. Identification of Inefficient Cycling Routes

**Figure 7:**
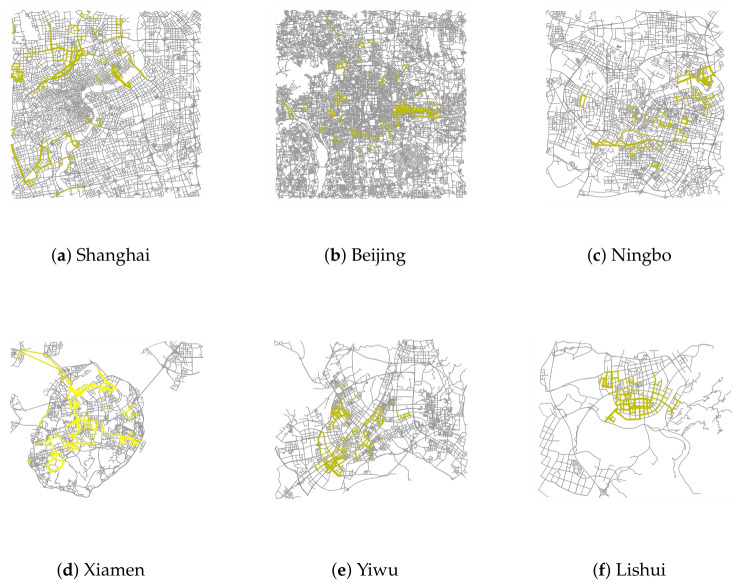
Cycling routes that require prior improvements in six cities, which are highlighted in yellow. Here, for the clarity of visualization, we show the top one hundred most inefficient routes in each city.

Apart from traffic analysis, the cycling-traffic-weighted-DR (i.e., $\frac{T_{ij}}{T}\times \frac{r_{ij}}{d_{ij}}$) can be applied to detect inefficient biking routes. When a route is more detoured, it is generally not friendly to cycling, as it would be harder to navigate [33], and when many people are using such routes to commute or connect commuting, then it should be improved with a higher priority. Here, via OSMnx (version 2.1.0) [48], we visualize the first one hundred inefficient cycling routes with the largest cycling-traffic-weighted-DR in each city (see Figure 7). In large cities (e.g., Shanghai, Beijing), many inefficient routes are located at the fringe of cities, where infrastructure and public transportation might be relatively underdeveloped compared to in the downtown area. Complex geographic or hydrological factors may also lead to inefficient routes, for example, the ones on the opposite sides of the Huangpu River in Shanghai, and the Dongyang River in Yiwu, or separated by Yundang Lake and Wanshi Mountain in Xiamen. Apart from segmenting effects posed by natural factors, human-made infrastructures might also pose similar effects. Wide roads might lead to “n”-shaped long detours when the origin and destination locations are on the opposite side (e.g., near the CBD of Beijing that is located around the East 3rd Ring Road to East 4th Ring Road, where travel demands posed by the working population there are also high). Some large street blocks (e.g., large gated working units, residential communities, universities, or even airports) also lead to encircled long detours (e.g., near Ningbo university, where the roads are blocked by a massive gated group of buildings, and in the Lishui downtown area, where dense residential communities are located). Compared to opening up gated regions for vehicle traffic, it might be viable to establish a biking-traffic-only lane through them (e.g., working units, residential communities [44], and universities). In addition, most identified efficient routes match with practical experience and observations. For example, in Xiamen, the top one hundred most inefficient routes identified by our metric are highly consistent with mountain–sea spatial trails and infrastructure-constrained routes. In the future, large-scale user surveys and systematic expert evaluation can be conducted for further validations.

## 6. Conclusions and Discussion

In this work, we first discover that the commonly applied detour ratio alone cannot effectively reflect the congestion level at the route level. Cycling flow, motor vehicle traffic, and road structure all play a part in traffic congestion, which is further validated with related data from Beijing. In addition, the cycling-traffic-weighted detour ratio can also be used to prescreen potentially inefficient routes for cycling, which are mainly caused by enclosed large regions, ineffective development of the infrastructure network, and segmentation effects posed by wide roads. Such inefficient cycling routes would be worth closer examination in urban and transportation planning processes. Our findings contribute to gaining a better understanding of the spatial patterns of cycling activities and cycling infrastructure that can help the transition towards a greener and more sustainable urban transportation.

However, several limitations remain. First of all, public transit also plays an important role in urban congestion, but it is excluded from this study due to inaccessibility. When such data is available, it should be integrated for a more comprehensive study. Second, we assume that cyclists prefer the fastest route between origin and destination locations; however, there can be multiple routes between locations and the route choice of cyclists can be affected by many other factors apart from traveling distance, including whether there are dedicated cycling lanes, the slope of roads, and perceived safety. In the future, with more detailed cycling trajectory data, we can obtain a more accurate cycling-traffic-weighted-detour ratio for each route. Third, apart from practical experience and observations, the identified inefficient routes can be better validated with large-scale surveys and systematic expert evaluation.

Possible further research includes the following aspects. Currently, most state-of-the-art collective mobility models [49,50,51] neglect the impacts of travel modes. Better understanding the generating mechanics behind cycling traffic and proposing collective mobility models tailored for cycling are worth closer investigations, as they will be crucial for predicting the evolution of the system after improving certain inefficient routes. In addition, although bikes have been the most affordable transportation conveyance, the influence of income on cycling activities via the dockless sharing bike can still be obvious at the collective level [15], which is worth future investigations for its impacts on transportation systems. There is a common concern that the expansion of fast transportation networks might bring about gentrification and further chase away the poor [52]. A better managed cycling infrastructure network [53,54] would be an important component towards a more sustainable and equal transportation system [37].

## Figures and Tables

**Table 1:** Basic statistics of datasets. The number of users and bikes and the size of the urban population are in millions, the urban area is in km^2^, and the GDP is in billions of RMB.

City	Platform	#Orders	Period (MM/DD,YY)	#Users	#Bikes	Population	Area	GDP
Shanghai	Mobike	1.02	08/01–08/31, 2016	0.017	0.300	24.89	6340.00	4470
Beijing	Mobike	3.20	05/10–05/24, 2017	0.350	0.485	21.89	16,455.13	4160
Ningbo	Hellobike	1.62	09/14–09/27, 2020	0.256	0.049	4.47	1147.76	1570
Xiamen	Aggregated	0.22	12/21–12/25, 2020	NA	0.053	2.11	192.74	780
Yiwu	Hellobike	0.39	09/14-09/27, 2020	0.089	0.021	1.89	575.66	184
Lishui	Hellobike	0.18	09/14–09/27, 2020	0.031	0.014	0.57	225.50	183

**Table 2:** Statistical distribution of the median detour ratio for cycling trips in six cities.

Time	Shanghai	Beijing	Ningbo	Xiamen	Yiwu	Lishu
Peak	1.33	1.56	1.29	1.42	1.30	1.28
Nonpeak	1.33	1.59	1.29	1.40	1.30	1.29

## Data Availability

The original datasets necessary to reproduce the results in the manuscript are available from the website of the Soda competition (http://shanghai.sodachallenges.com/data.html?lang=en, accessed on 1 May 2023) for the Shanghai dataset, and Mobike Cup competition (https://biendata.com/competition/mobike/data/, accessed on 1 May 2023) for the Beijing dataset. The Xiamen data is accessible upon reasonable request. The original datasets of Ningbo, Yiwu and Lishui are not publicly available due to the commercially sensitive information contained and the non-disclosure agreement with HelloBike. The codes will be available upon reasonable request to the authors.

## Appendix A. Cycling Duration Distribution

The distributions of cycling duration peak roughly between 3 and 6 min, but also exhibit power-law fat tails [55] across cities (see Figure A1). This suggests that the average cycling duration is not as representative as ordinarily assumed [38], and there is a notable fraction of trips of a much longer cycling duration than the average when compared to a Poisson or Normal distribution with the same mean value. This is interesting given the fact that during rush hours, there are more longer distance trips.

Note that the Beijing dataset does not provide the actual arrival time of each trip; we make estimations based on expected cycling durations queried from the Amap API (https://lbs.amap.com/api/webservice/guide/api/direction, accessed on 1 May 2023) for the shortest route between given origin and destination locations, which will be trivially proportional to the routing distance. Interestingly, the traveling characteristics of cycling activities are contrary to those taking taxis. The taxi tends to have a shorter trip distance and duration during rush hours than during nonpeak hours [36].

## Appendix B. Road Width Distribution

We use the number of vehicle lanes of a road to approximate its width (see Figure A2). The road networks for Shanghai, Beijing, Ningbo, Xiamen, Yiwu and Lishui are obtained from OpenStreetMap [47] via the OSMnx [48] in Python 3.8. Each road is associated with a variety of metadata, including the category of the road, the speed limit and the number of lanes. Here, we consider roads in the downtown area, which covers the region of cycling activities.

## References

[1] Jacobs J. (1961). The Death and Life of Great American Cities.

[2] WHO (2002). A Physically Active Life Through Everyday Transport with a Special Focus on Children and Older People and Examples and Approaches from Europe.

[3] DeMaio P. (2009). Bike-sharing: History, impacts, models of provision, and future. J. Public Transp..

[4] Jäppinen S., Toivonen T., Salonen M. (2013). Modelling the potential effect of shared bicycles on public transport travel times in Greater Helsinki: An open data approach. Appl. Geogr..

[5] Diao M., Kong H., Zhao J. (2021). Impacts of transportation network companies on urban mobility. Nat. Sustain..

[6] Li R., Dong L., Zhang J., Wang X., Wang W.X., Di Z., Stanley H.E. (2017). Simple spatial scaling rules behind complex cities. Nat. Commun..

[7] Dill J., Carr T. (2003). Bicycle commuting and facilities in major US cities: If you build them, commuters will use them. Transp. Res. Rec..

[8] Hull A., O’Holleran C. (2014). Bicycle infrastructure: Can good design encourage cycling?. Urban Plan. Transp. Res..

[9] Life B. (2019). Transforming Cities: The Potential of Everyday Cycling.

[10] Cheng L., Mi Z., Coffman D., Meng J., Liu D., Chang D. (2021). The Role of Bike Sharing in Promoting Transport Resilience. Netw. Spat. Econ..

[11] Teixeira J.F., Lopes M. (2020). The link between bike sharing and subway use during the COVID-19 pandemic: The case-study of New York’s Citi Bike. Transp. Res. Interdiscip. Perspect..

[12] Jiang H., Song S., Zou X., Lu L. (2020). How Dockless Bike Sharing Changes Lives: An Analysis of Chinese Cities.

[13] Li R., Richmond P., Roehner B.M. (2018). Effect of population density on epidemics. Phys. A Stat. Mech. Its Appl..

[14] Glick D.M., Einstein K.L., Palmer M., Fox S. (2020). 2020 Menino Survey: COVID-19 Recovery and the Future of Cities.

[15] Yang Y., Gao T., Xu Z., Liu C., Yang Y., Shang F., Li R. (2024). Quantifying relation between mobility patterns and socioeconomic status of dockless sharing-bike users. Int. J. Mod. Phys. C.

[16] Li R., Gao S., Luo A., Yao Q., Chen B., Shang F., Jiang R., Stanley H.E. (2021). Gravity model in dockless bike-sharing systems within cities. Phys. Rev. E.

[17] Sun Y. (2018). Sharing and riding: How the dockless bike sharing scheme in China shapes the city. Urban Sci..

[18] Li R., Bai T., Luo A., Yang Y., Lü L., Fan J., Zhang Y., Lu G., Stanley H.E. (2025). Emergence of scaling in dockless bike-sharing systems for bike choice behavior. J. Phys. Complex..

[19] Martin E.W., Shaheen S.A. (2014). Evaluating public transit modal shift dynamics in response to bikesharing: A tale of two US cities. J. Transp. Geogr..

[20] Shaheen S.A., Cohen A.P., Martin E.W. (2013). Public bikesharing in North America: Early operator understanding and emerging trends. Transp. Res. Rec..

[21] Cavill N., Kahlmeier S., Racioppi F. (2006). Physical Activity and Health in Europe: Evidence for Action.

[22] Rojas-Rueda D., De Nazelle A., Tainio M., Nieuwenhuijsen M.J. (2011). The health risks and benefits of cycling in urban environments compared with car use: Health impact assessment study. BMJ.

[23] Shaheen S.A., Guzman S., Zhang H. (2010). Bikesharing in Europe, the Americas, and Asia: Past, present, and future. Transp. Res. Rec..

[24] Gössling S., Schröder M., Späth P., Freytag T. (2016). Urban space distribution and sustainable transport. Transp. Rev..

[25] Szell M. (2018). Crowdsourced quantification and visualization of urban mobility space inequality. Urban Plan..

[26] Hamilton T.L., Wichman C.J. (2018). Bicycle infrastructure and traffic congestion: Evidence from DC’s Capital Bikeshare. J. Environ. Econ. Manag..

[27] Fishman E., Washington S., Haworth N. (2013). Bike share: A synthesis of the literature. Transp. Rev..

[28] Noland R.B., Ishaque M.M. (2006). Smart bicycles in an urban area: Evaluation of a pilot scheme in London. J. Public Transp..

[29] Yang H., Ke J., Ye J. (2018). A universal distribution law of network detour ratios. Transp. Res. Part C Emerg. Technol..

[30] Dong L., Li R., Zhang J., Di Z. (2016). Population-weighted efficiency in transportation networks. Sci. Rep..

[31] Cardillo A., Scellato S., Latora V., Porta S. (2006). Structural properties of planar graphs of urban street patterns. Phys. Rev. E.

[32] Gastner M.T., Newman M.E. (2006). Shape and efficiency in spatial distribution networks. J. Stat. Mech. Theory Exp..

[33] Coutrot A., Manley E., Goodroe S., Gahnstrom C., Filomena G., Yesiltepe D., Dalton R., Wiener J., Hölscher C., Hornberger M. (2022). Entropy of city street networks linked to future spatial navigation ability. Nature.

[34] De Fabiis F., Baldini M., Coppola P. (2026). The impact of geographical context on Bikeability perception: A gender-difference analysis. J. Transp. Geogr..

[35] Arellana J., Saltarín M., Larrañaga A.M., Alvarez V., Henao C.A. (2020). Urban walkability considering pedestrians’ perceptions of the built environment: A 10-year review and a case study in a medium-sized city in Latin America. Transp. Rev..

[36] Feng X., Sun H., Gross B., Wu J., Li D., Yang X., Lv Y., Zhou D., Gao Z., Havlin S. (2022). Scaling of spatio-temporal variations of taxi travel routes. New J. Phys..

[37] Liu Q., Chen C.L., Cao M. (2021). Exploring the relationship between the commuting experience and hedonic and eudaimonic well-being. Transp. Res. Part D Transp. Environ..

[38] Chen J., Zhang Y., Zhang R., Cheng X., Yan F. (2019). Analyzing users’ attitudes and behavior of free-floating bike sharing: An investigating of Nanjing. Transp. Res. Procedia.

[39] Bettencourt L.M., Lobo J., Helbing D., Kühnert C., West G.B. (2007). Growth, innovation, scaling, and the pace of life in cities. Proc. Natl. Acad. Sci. USA.

[40] Liu C., Yang Y., Chen B., Cui T., Shang F., Fan J., Li R. (2022). Revealing spatio-temporal interacting patterns behind complex cities. Chaos.

[41] Black W.R. (2003). Transportation: A Geographical Analysis.

[42] Barthélemy M. (2011). Spatial networks. Phys. Rep..

[43] Louf R., Barthelemy M. (2014). A typology of street patterns. J. R. Soc. Interface.

[44] Li L., Wan W.X., He S. (2021). The Heightened ‘Security Zone’Function of Gated Communities during the COVID-19 Pandemic and the Changing Housing Market Dynamic: Evidence from Beijing, China. Land.

[45] Çolak S., Lima A., González M.C. (2016). Understanding congested travel in urban areas. Nat. Commun..

[46] Xu Y., Li R., Jiang S., Zhang J., González M.C. Clearer skies in Beijing–revealing the impacts of traffic on the modeling of air quality. Proceedings of the 96th Annual Meeting Transportation Research Board.

[47] (2022). OpenStreetMap. https://www.openstreetmap.org.

[48] Boeing G. (2017). OSMnx: New methods for acquiring, constructing, analyzing, and visualizing complex street networks. Comput. Environ. Urban Syst..

[49] Simini F., González M.C., Maritan A., Barabási A.L. (2012). A universal model for mobility and migration patterns. Nature.

[50] Yan X.Y., Zhao C., Fan Y., Di Z., Wang W.X. (2014). Universal predictability of mobility patterns in cities. J. R. Soc. Interface.

[51] Yan X.Y., Wang W.X., Gao Z.Y., Lai Y.C. (2017). Universal model of individual and population mobility on diverse spatial scales. Nat. Commun..

[52] Kahn M.E. (2007). Gentrification trends in new transit-oriented communities: Evidence from 14 cities that expanded and built rail transit systems. Real Estate Econ..

[53] Olmos L.E., Tadeo M.S., Vlachogiannis D., Alhasoun F., Alegre X.E., Ochoa C., Targa F., González M.C. (2020). A data science framework for planning the growth of bicycle infrastructures. Transp. Res. Part C Emerg. Technol..

[54] Szell M., Mimar S., Perlman T., Ghoshal G., Sinatra R. (2022). Growing urban bicycle networks. Sci. Rep..

[55] Brockmann D., Hufnagel L., Geisel T. (2006). The scaling laws of human travel. Nature.

